# Defective tumor necrosis factor release from Crohn's disease macrophages in response to toll-like receptor activation: Relationship to phenotype and genome-wide association susceptibility loci

**DOI:** 10.1002/ibd.22952

**Published:** 2012-03-20

**Authors:** Gavin W Sewell, Farooq Z Rahman, Adam P Levine, Luke Jostins, Philip J Smith, Ann P Walker, Stuart L Bloom, Anthony W Segal, Andrew M Smith

**Affiliations:** 1 Division of Medicine, University College LondonLondon, UK; 2 Department of Gastroenterology, UCLH NHS Foundation TrustLondon, UK; 3 Statistical and Computational Genetics, Wellcome Trust Sanger Institute, Wellcome Trust Genome CampusCambridge, UK

**Keywords:** IBD, inflammation, genetic polymorphisms, macrophages, Crohn's disease

## Abstract

**Background:**

Recent work provides evidence of a failure of acute inflammation in Crohn's disease (CD), and suggests that the primary defect operates at the level of the macrophage and cytokine release. Here we extend the characterization of the innate immune defect in CD by investigating the macrophage response to Toll-like receptor (TLR) agonists and assess potential links between genome-wide association study (GWAS) susceptibility loci, disease phenotype, and therapeutic regimens on tumor necrosis factor α (TNF) release.

**Methods:**

Peripheral blood-derived macrophages were cultured from control subjects and patients with CD, stimulated with TLR ligands, and the release of TNF measured. Genomic DNA was purified from blood and genotyped for 34 single nucleotide polymorphisms (SNPs) identified as being associated with CD by GWAS.

**Results:**

All stimuli resulted in a reduction (32%–48%) in TNF release from macrophages derived from CD patients (*n* = 28–101) compared to those from healthy control (HC) subjects. All phenotypes demonstrated impaired TNF release, with the greatest defect in patients with colonic disease. There was no detectable relationship between the level of TNF released and the presence of GWAS susceptibility loci in CD patients. Reduced TNF levels were not influenced by age, gender, or use of aminosalicylate (5-ASA) medication.

**Conclusions:**

This study supports the hypothesis of defective proinflammatory cytokine secretion and an innate immunodeficiency in CD. Abnormal TNF secretion is evident downstream of multiple TLRs, affects all disease phenotypes, and is unrelated to 34 polymorphisms associated with CD by GWAS. (Inflamm Bowel Dis 2012;)

Crohn's disease (CD) is a chronic relapsing inflammatory disease of the gastrointestinal tract associated with considerable lifelong morbidity.^1^ It is characterized by granulomatous inflammation that most frequently affects the terminal ileum and colon. The incidence of CD has risen dramatically since the latter part of the 20th century for reasons that remain poorly understood.^2^

A number of genetic and environmental factors have been associated with CD. Recently, genome-wide association studies (GWAS) have identified a number of single nucleotide polymorphisms (SNPs) with significantly different allele frequencies between CD and healthy control (HC) cohorts. It has been estimated that these susceptibility variants account for ≍23% of the total heritability of the disease. Some of the strongest associations include *NOD2*, the interleukin (IL)-23 receptor *IL-23R*, and two genes with roles in autophagy (*ATG16L1* and *IRGM*).^3^, ^4^ Many of the CD-associated genes identified to date appear to have important roles in sensing, clearance, and propagation of the inflammatory response to commensal microbiota. However, although many of the loci are suspected to influence immune system function, the functional variants within each locus and the underlying pathogenic mechanism remain to be elucidated.

Mounting evidence suggests that CD may result from an innate immunodeficiency.^5–7^ Although the precise mechanisms responsible for this abnormality remain to be determined, a primary pathogenic defect in the macrophage response to bacteria has recently been shown. Macrophages, the sentinels of the immune system, orchestrate cellular responses against a complex and diverse range of intestinal microbial insults via pattern recognition receptors such as Toll-like receptors (TLRs) and NOD-like receptors (NLRs).^8^, ^9^ Macrophage activation through these and other interconnected but subtly distinct signaling pathways induces secretion of inflammatory mediators that recruit and activate other leukocytes from the surrounding microcirculation. It is postulated that the dysregulated macrophage response to bacteria is central to the pathogenesis of CD. A number of recent studies have demonstrated impaired levels of macrophage proinflammatory cytokine release, including tumor necrosis factor-α (TNF), in response to *E. coli* stimulation.^7^, ^10^ Attenuated macrophage proinflammatory cytokine secretion may result in the impaired neutrophil recruitment and bacterial clearance observed in patients with CD. The retention of the undigested bacteria at sites of ingress was proposed to be the driving force for the ensuing chronic inflammatory reaction and granulomatous pathology that characterizes CD.^6^, ^7^

TLRs are transmembrane pathogen pattern recognition receptors expressed by myelomonocytic and epithelial cells.^11^ Each TLR recognizes specific bacterial or viral components; key TLRs in the response to bacteria include TLR4, which recognizes lipopolysaccharide (LPS), TLR2, which recognizes lipoteichoic acid, peptidoglycan and the synthetic tripalmitoylated lipopeptide PAM_3_CSK_4_, and TLR5, which binds to flagellin.^12^ Ligand-receptor engagement results in intracellular signaling cascades and the induction of effector responses important for the innate immune defense against microbes. The potential relevance of TLRs in the response to intestinal microbiota is demonstrated by TLR4 knockout mice, which have earlier and more pronounced gastrointestinal bleeding than wildtype mice after administration of dextran sodium sulfate (DSS), which coincided with increased bacterial translocation and impaired neutrophil recruitment.^13^ Differential expression of TLRs have been documented in biopsy samples from inflammatory bowel disease (IBD) patients.^14^, ^15^ A number of studies have reported associations between TLR4 polymorphisms and CD,^16^ although these were not replicated in a recent large GWAS meta-analysis.^4^

TNF, a potent mediator of inflammation and major target of biological therapy in CD, was chosen as the focus of this study to assess the acute inflammatory response of macrophages following microbial stimulation. In the present study we compared TNF release from CD and HC macrophages after TLR2, TLR4, and TLR5 activation. In addition, we used these results in combination with patient information and SNP genotypes to test for any association between defective proinflammatory responses, inheritable risk factors, and disease phenotype.

## MATERIALS AND METHODS

### Patients

Adult patients with definitive diagnoses of CD confirmed using standard diagnostic criteria were recruited from the Gastroenterology Outpatient Clinic at University College London Hospitals NHS Foundation Trust (UCLH). None of the patients studied showed any clinical or biochemical evidence of impaired nutritional state. In some experiments, patients with ulcerative colitis (UC), a clinically and histopathologically distinct form of IBD, were used as a control group in addition to healthy subjects. All patients recruited had Harvey–Bradshaw scores of <3 or partial Mayo score <3 for CD and UC, respectively, both of which have been validated for assessment of disease activity.^17^, ^18^ All individuals were on no medication or on maintenance 5-aminosalicylic acid (5-ASA) at the time of sample collection (Table 1).

**Table 1:** Demographics of the Subjects

	HC	CD	UC
*n*	41	101	47
Gender (M:F)	21:20	37:64	24:23
Mean age	32.8	41.6	44.5
Age standard deviation	9.93	15.3	15.9
Age range	20-59	18-75	21-75
Smokers	5	20	3
Treatment			
No medication		25	
5-ASA only		76	
Others		0	
Disease location			
L1		32	
L2		44	
L3		24	
L4		1	
Genotype information	36	97	

Disease location was subdivided into ileal (L1), colonic (L2), ileocolonic (L3), and upper gastrointestinal (L4).

These studies were approved by the Joint UCL/UCLH Committees on the Ethics of Human Research (02/0324). Written informed consent was obtained from all volunteers. No patient was studied more than once in each of the different sets of experiments.

### Macrophage Isolation, Culture, and Stimulation

Peripheral venous blood samples were collected from subjects into heparinized syringes (5 U/mL). Mononuclear cells were isolated by differential centrifugation (900*g*, 30 minutes, 20°C) over Lymphoprep (Axis-Shield, Oslo, Norway) and washed twice with sterile phosphate-buffered saline (PBS) (Gibco, Paisley, UK) at 300*g* (5 minutes, 20°C). Cells were resuspended in 10 mL RPMI-1640 medium (Invitrogen, Paisley, UK) supplemented with 100 U/mL of penicillin (Gibco) and 100 μg/mL streptomycin (Gibco) and 20 mM Hepes buffer (Sigma-Aldridge, Poole, UK) (RPMI), and plated at a density of ≍5 × 10^6^ cells/mL in 8 cm^2^ Nunclon Surface tissue culture dishes (Nunc, Roskilde, Denmark). After an initial culture period of 2 hours at 37°C, 5% CO_2_, the nonadherent cells were discarded and 10 mL of fresh RPMI supplemented with 10% fetal bovine serum (Sigma) (10% FBS/RPMI) added to each tissue culture dish. Cells were then cultured for 5 days at 37°C, 5% CO_2_, with the addition of a further 10 mL fresh 10% FBS/RPMI after 24 hours.

Adherent cells were scraped on day 5 and replated in 96-well culture plates at equal densities (10^5^/well) in X-Vivo-15 medium (Cambrex, Walkersville, MD). These primary monocyte-derived macrophages were incubated overnight at 37°C, 5% CO_2_ to adhere, and then stimulated for up to 24 hours 200 ng/mL LPS (Alexis, San Diego, CA), 2 μg/mL Pam_3_-Cys-Lys_4_ (Alexis) and 20 ng/mL flagellin (Alexis).

### TNF Release After TLR Stimulation

TNF release was measured using a cytotoxicity bioassay (obtained from Prof. B. Beutler, Scripps Institute, La Jolla, CA) as previously described.^19^ Murine L929 fibroblast cells were grown in Dulbecco's modified Eagle's medium (DMEM; Gibco), supplemented with 10% FBS (Sigma), 100 U/mL of penicillin (Gibco), and 100 μg/mL streptomycin (Gibco), at 37°C, 5% CO_2_. A confluent monolayer of murine L929 fibroblasts was trypsinized and resuspended to 4 × 10^5^ cells/mL in DMEM. L929 cells were seeded into 96-well flat-bottom tissue culture plates (4 × 10^4^ cells/well) and incubated overnight at 37°C, 5% CO_2_. After overnight culture the medium was discarded, replaced by 50 μL DMEM containing cycloheximide (0.04 mg/mL), and incubated for 20 minutes at 37°C, 5% CO_2_. Fifty μL of cell-free supernatant (diluted 1:50 in DMEM), collected from primary macrophages as described, was added to individual wells. Serially diluted recombinant human TNF (R&D Systems, Minneapolis, MN) (100-0 pg/mL) was used to determine the standard curve for the assay.

Cytokine release in culture supernatants was normalized for the numbers of viable cells in each well, ascertained with the MTT (3-[4,5-dimethylthiazol-2-yl]-2,5-diphenyl tetrozolium bromide, tetrazolium salt) assay (Boehringer Ingelheim, Berkshire, UK). Twenty μL of 2.5 ng/mL MTT was added to each well and incubated for 4 hours at 37°C, 5% CO_2_. Supernatants were carefully discarded and 100 μL/well of lysis solution (90% isopropanol, 0.5% sodium dodecyl sulfate [SDS], 0.04 N HCl, 10% H_2_0) added to each well for 1 hour at room temperature. The absorbance was read at 570 nm using a microplate reader (Anthos Labtec Instruments, Salzburg, Austria).

### Purification of Total Genomic DNA

Peripheral venous blood samples were collected from subjects into heparinized syringes (5 U/mL) and genomic DNA prepared, using the QIAamp DNA blood Mini Kit (Qiagen, Hilden, Germany). Optical density readings were determined for OD_260_/OD_280_ and OD_260_/OD_230_ using a NanoDrop ND-1000 spectrophotometer (Fisher Scientific, Loughborough, UK) to assess protein and solvent contamination, respectively.

### SNP Genotyping

Genotyping for 34 CD-associated SNPs was performed using the iPLEX Gold Assay (Sequenom, San Diego, CA), shown in Supporting Table 1. The SNPs were selected based on the results of previous GWAS.^3^ Assays for all SNPs were designed using the eXTEND suite and MassARRAY Assay Design software v. 3.1 (Sequenom). Amplification was conducted in a total volume of 5 μL containing ≍0.06–0.4 ng genomic DNA, 100 nM of each PCR primer, 500 μM of each dNTP, 1.25 × PCR buffer (Qiagen), 1.625 mM MgCl_2_, and 1U HotStar Taq (Qiagen). Reactions were heated to 94°C for 15 minutes followed by 45 cycles at 94°C for 20 seconds, 56°C for 30 seconds, and 72°C for 1 minute, then a final extension at 72°C for 3 minutes. Unincorporated dNTPs were shrimp alkaline phosphatase (SAP)-digested prior to iPLEX Gold allele specific extension with mass-modified ddNTPs using an iPLEX Gold reagent kit (Sequenom), in accordance with the manufacturer's instructions. Reaction extension primer concentrations were adjusted to between 0.7–1.8 μM, dependent on primer mass. Extension products were desalted and dispensed onto a SpectroCHIP using a MassARRAY Nanodispenser prior to matrix-assisted laser desorption ionization time-of-flight (MALDI-TOF) analysis with a MassARRAY Analyzer Compact mass spectrometer. Genotypes were automatically assigned and manually confirmed using MassARRAY TyperAnalyzer software v. 4.0 (Sequenom). In a few cases the genotypes could not be assigned and these individuals were therefore excluded from the analysis.

### Statistical Analysis

All data are presented as mean ± standard error of the mean (SEM). Statistical significance between groups was evaluated using a one-way analysis of variance (ANOVA) with Tukey posttest or an unpaired two-tailed Student's *t*-test when only two groups were compared. Mean differences were considered significant when *P* < 0.05. Correlation analysis was assessed using Pearson's correlation coefficient.

## RESULTS

### CD Macrophages Release Diminished TNF After TLR Stimulation

**Figure 1:**
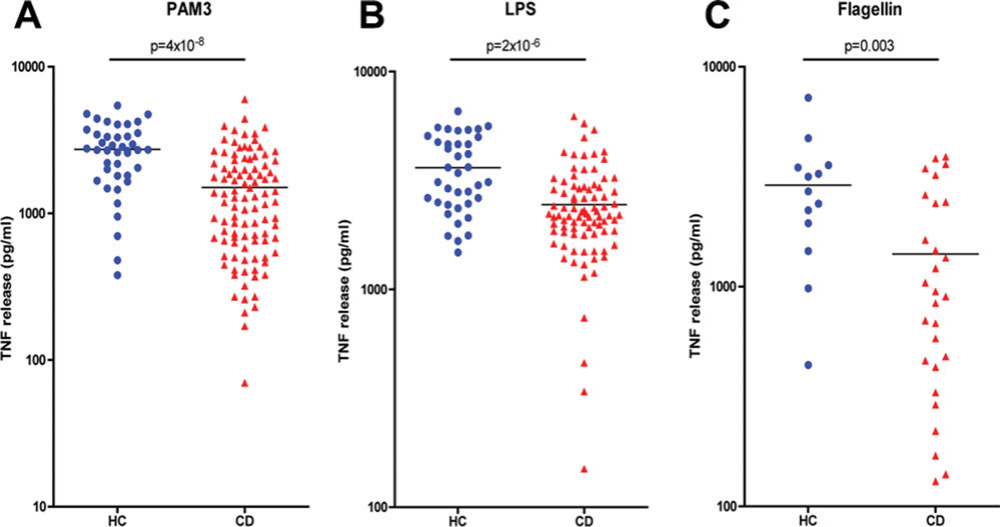
Macrophages from CD patients release attenuated TNF in response to TLR stimulation. Monocyte-derived macrophages stimulated for 6 hours with PAM_3_CSK_4_ (PAM3), LPS, and flagellin, and TNF levels in supernatants were quantified using the TNF bioassay. (A) TLR2 response was measured in macrophages from HC (*n* = 41) and CD (*n* = 101) after PAM3 stimulation. (B) TLR4 response was measured in macrophages from HC (*n* = 38) and CD (*n* = 87) after LPS stimulation. (C) TLR5 response was measured in macrophages from HC (*n* = 13) and CD (*n* = 28) after flagellin stimulation. TNF levels from each subject are depicted with the mean value shown as a black horizontal bar with *P*-value. Data are shown on a logarithmic scale. [Color figure can be viewed in the online issue, which is available at wileyonlinelibrary.com.]

We first determined whether macrophage TNF secretion was defective after various TLR stimuli. Monocyte-derived macrophages were activated with the TLR ligands PAM_3_CSK_4_ (HC *n* = 41 and CD *n* = 101), LPS (HC *n* = 33 and CD *n* = 83), and flagellin (HC *n* = 13 and CD *n* = 28). In all cases macrophages isolated from CD patients demonstrated grossly attenuated TNF secretion compared to control subjects (Fig. .1). TLR2 activation by PAM_3_CSK_4_ resulted in macrophages from CD patients releasing TNF at levels which were equivalent to a 45% reduction compared to the control group (*P* = 4 × 10^−8^). LPS exposure results in TLR4 activation and release of TNF from CD macrophages, and again this was significantly lower than the control group (32%, *P* = 2 × 10^−6^). Similarly, TLR5 ligation by flagellin demonstrated attenuation in TNF release (48%, *P* = 0.003). Reduced TNF secretion was not due to differences between CD and HC macrophage phenotype, reduced TLR expression, or abnormalities in receptor signaling and gene induction (Supporting Fig. 1).^7^ These results provide evidence to support an altered macrophage immune response to microbial challenge in CD and show that the defect is not restricted to an individual receptor.

### Association Between Aberrant TNF Release and Disease Phenotype

**Figure 2:**
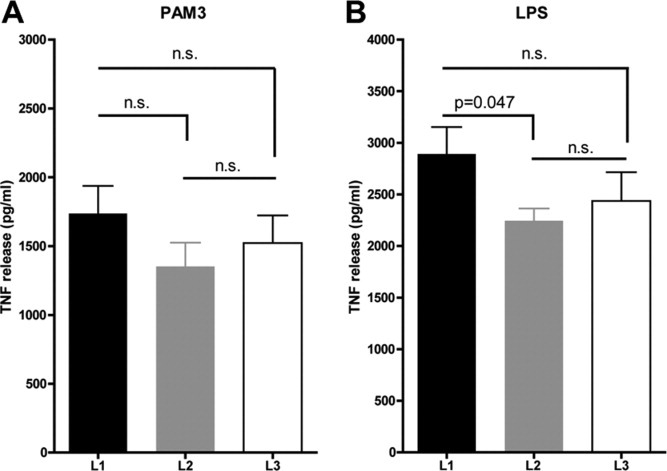
Defective TNF release is common to all phenotypes. CD subjects were divided into three phenotypic subgroups based on disease location, including ileal (L1, *n* = 32), colonic (L2, *n* = 44), and ileocolonic (L3, *n* = 24) involvement. (A) There were no significant differences in macrophage TNF release after TLR2 stimulation between disease phenotypes. (B) Macrophages from patients with colonic CD release significantly less TNF than ileal patients after TLR4 stimulation with LPS. Macrophages from all groups released significantly less TNF than the HC cohort in response to TLR2 and TLR4 stimulation. Results expressed as mean ± SEM with *P*-value.

It is now recognized that CD is a syndrome and can be divided into subtypes depending on disease phenotype, ileal (L1), colonic (L2), and ileocolonic (L3).^20^ It was therefore of interest to subdivide our CD patients into phenotypic groups and compare the levels of TNF released after PAM_3_CSK_4_ and LPS stimulation (Fig. .2). All three phenotypes demonstrated defective TNF secretion compared to HC after stimulation with either PAM_3_CSK_4_ or LPS. Direct comparison between all three CD phenotypes revealed that L1 patients released higher TNF levels than L2 patients and this difference reached significance with LPS (*P* = 0.047). Therefore, diminished TNF secretion is common to all three phenotypes and confirms our previous findings demonstrating defective TNF release after *E. coli* stimulation in both ileal and colonic patients.^7^

### Abnormal TNF Release Is Not Dependent on Prior Bowel Inflammation, Gender, Age, or Medication

**Figure 3:**
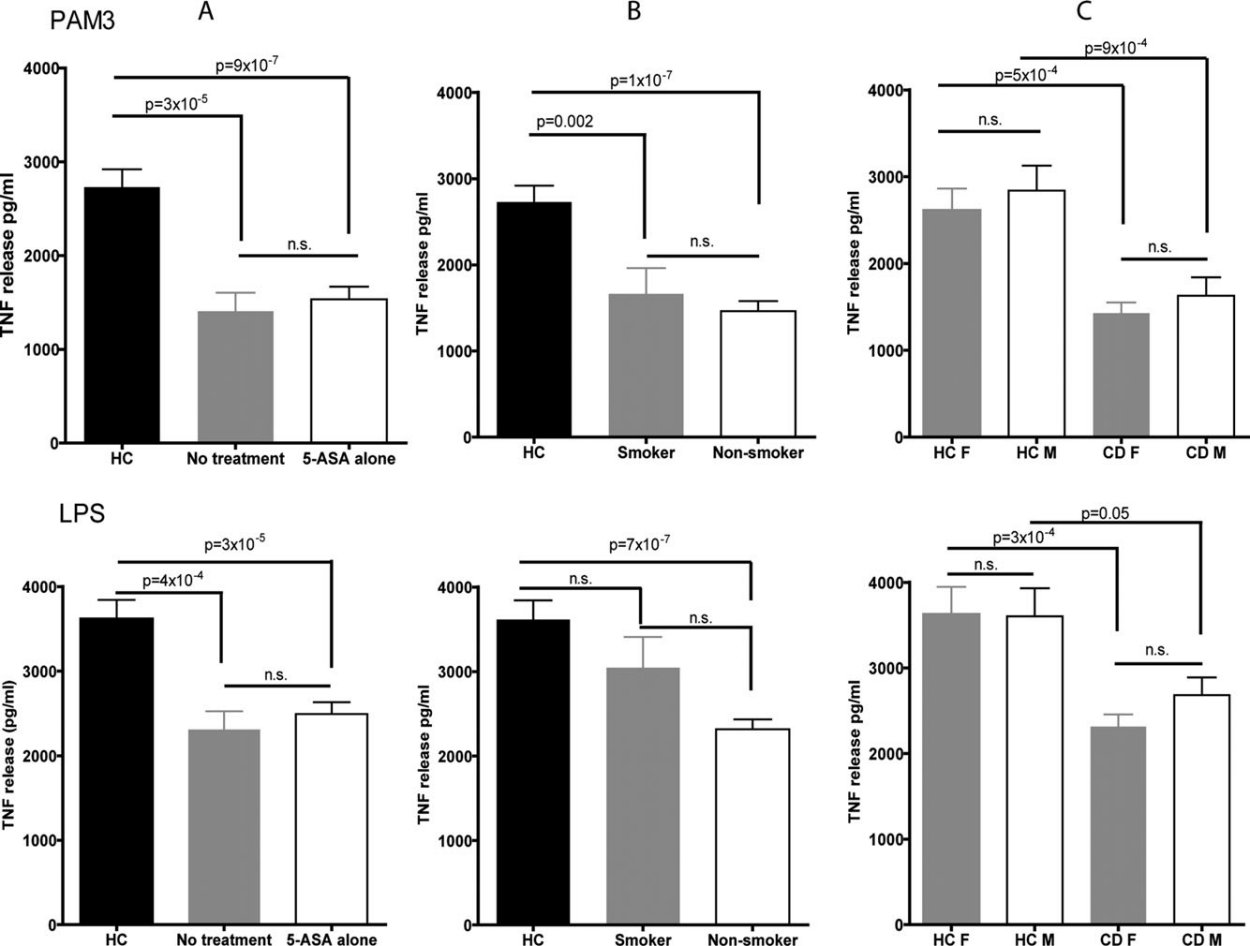
TNF secretion after TLR2 and TLR4 activation in relation to medical treatment, smoking, and gender. CD patients and where applicable HC were divided by (A) treatment (CD no-treatment *n* = 25, 5-ASA treatment *n* = 76; HC *n* = 38), (B) smoking (CD smoker *n* = 20, nonsmoker *n* = 81), and (C) gender, (CD male [M] *n* = 37, female [F] *n* = 64; HC male [M] *n* = 21, female [F] *n* = 20). All results are expressed as mean ± SEM with *P*-value.

We examined whether factors such as prior bowel inflammation, medication, smoking tobacco, age, and gender could have a confounding influence on macrophage TNF secretion in response to TLR agonists. No correlation was observed between age and TNF release after either PAM_3_CSK_4_ (*r*^2^ = 0.005, *P* = 0.94) or LPS (*r*^2^ = 0.0164, *P* = 0.24) stimulation (Supporting Fig. 2). In addition, there was no association between current medication, smoking status, or gender (Fig. .3A–C). Interestingly, CD patients who smoked released TNF levels that were not significantly different from HC after LPS stimulation; however, the same individuals did demonstrate impaired TNF secretion after PAM_3_CSK_4_ exposure (*P* = 0.002).

**Figure 4:**
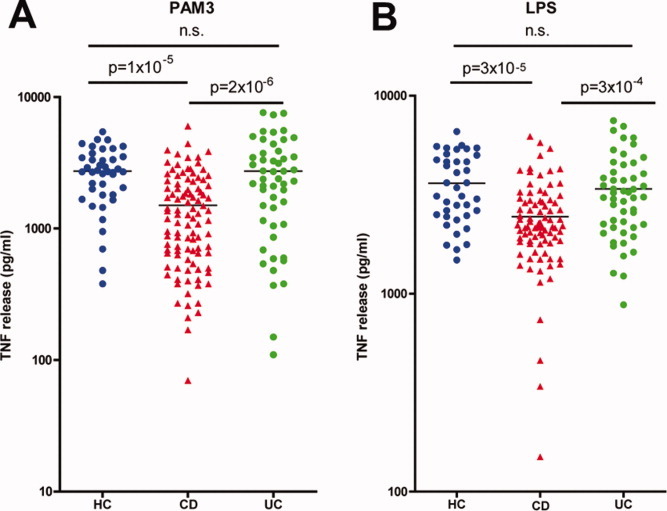
Macrophages from UC patients release normal levels of TNF in response to TLR stimulation. Supernatants from macrophages stimulated for 6 hours with PAM3 and LPS were tested for the levels of TNF released. (A) TLR2 response was measured in macrophages from HC (*n* = 41), CD (*n* = 101), and UC (*n* = 47) after PAM3 stimulation. (B) TLR4 response was measured in macrophages from HC (*n* = 38), CD (*n* = 87), and UC (*n* = 47) after LPS stimulation. TNF levels from each subject are depicted with the mean value shown as a black horizontal bar with *P*-value. [Color figure can be viewed in the online issue, which is available at wileyonlinelibrary.com.]

In order to determine whether previous bowel inflammation could influence macrophage TNF release, patients with UC were investigated as an additional control cohort. Macrophages from UC subjects (*n* = 47) released TNF at levels equivalent to HC, and significantly greater than CD patients after PAM_3_CSK_4_ (*P* = 2 × 10^−6^) and LPS stimulation (*P* = 3 × 10^−4^) (Fig. .4).

These results demonstrate that the attenuated TNF release by CD macrophages is attributable to the disease and independent of previous bowel inflammation, use of 5-ASA medication, age, or gender. Smoking status seems to be more complex, with an apparent normal TNF release downstream of TLR4 in smokers coinciding with a defective TLR2 response. Further investigation will be necessary to determine the mechanism of these observations.

### Diminished TNF Secretion Is Not Dependent on CD Susceptibility Polymorphisms

**Figure 5:**
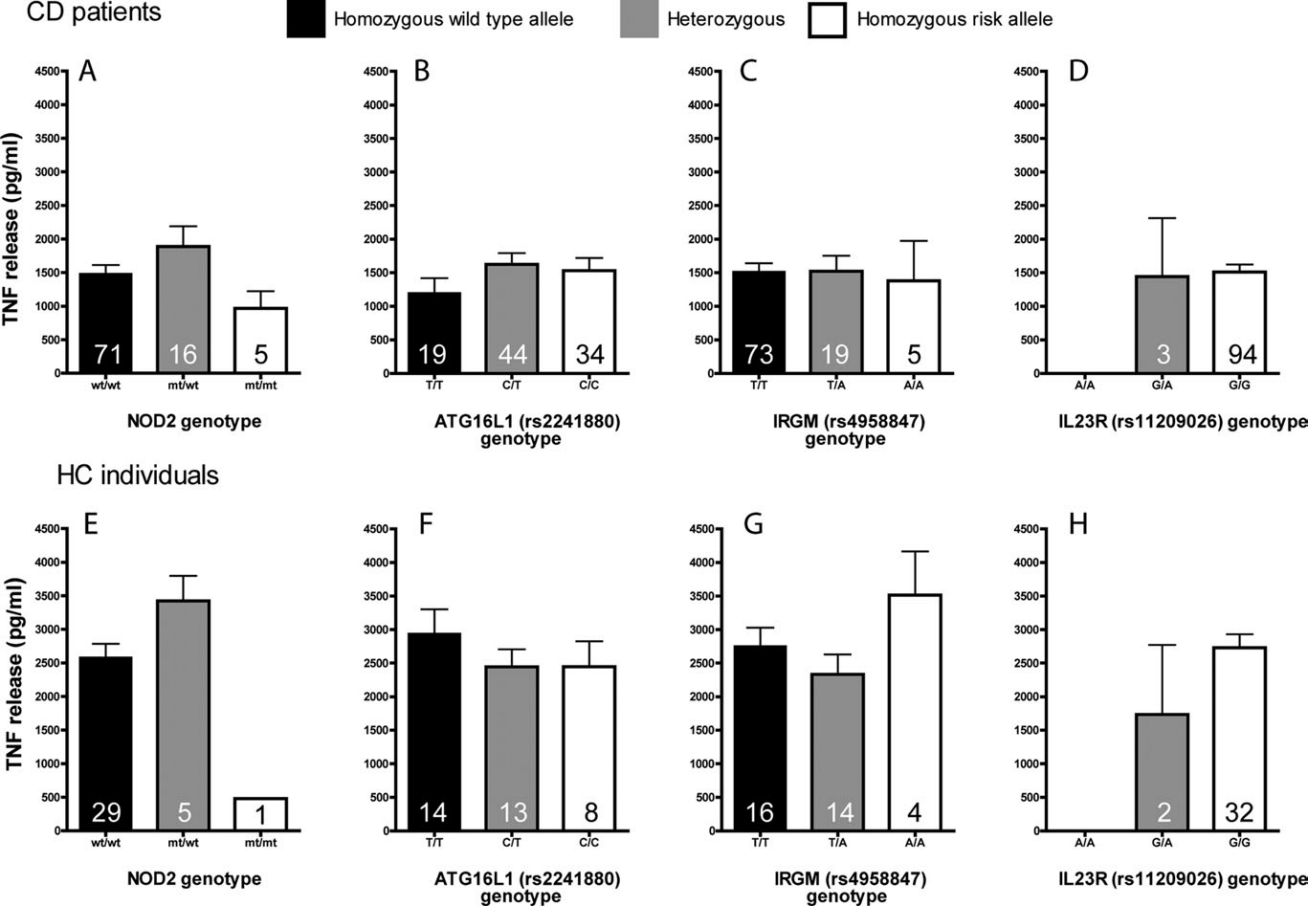
Relationship between TNF secretion after TLR2 activation and GWAS SNPs. Patients were typed for 34 SNPs associated with increased risk of developing CD and separated into homozygous nonrisk (black), heterozygous (gray), and homozygous risk (white) for (A) *NOD2* (compound heterozygotes where grouped with homozygous risk), (B) *IRGM*, (C) *ATG16L1*, and (D) *IL23R* and the corresponding TNF levels after TLR2 activation shown. Corresponding results for the HC cohort are shown in (E–H). Results expressed as mean ± SEM with *P*-value, value shown in each bar represents the number of individuals in each genotype.

GWAS have identified over 70 loci that are associated with CD.^3^, ^4^ In this study we looked for association between 34 SNPs strongly associated with CD and TNF secretion downstream of TLR2 (Fig. .5; Supporting Table 1). *NOD2* is the strongest CD-associated gene identified to date, which encodes an intracellular sensor of muramyl dipeptide (MDP). The three CD-associated polymorphisms in *NOD2* have been shown to result in defective pro- and antiinflammatory cytokine induction after stimulation with MDP.^21^ We investigated the effect *NOD2* polymorphisms had on TNF release downstream of TLR2 stimulation. Dividing our CD cohort into patients either homozygous or compound heterozygous (*n* = 5), heterozygous (*n* = 16), or wildtype (*n* = 71) for *NOD2* polymorphisms showed no significant effect on TNF release after PAM_3_CSK_4_ stimulation (Fig. .5A). In addition, the attenuated TNF release was independent of polymorphisms in the autophagy-associated genes *ATG16L1* and *IRGM* (Fig. .5B,C), and *IL-23R* (Fig. .5D). The other 28 SNPs tested also demonstrated no effect on TNF release in HC and CD cohorts (data not shown). The same findings were also evident after TLR4 stimulation (data not shown). HC who carry one *NOD2* CD risk allele release levels of TNF equivalent to individuals with two wildtype alleles, whereas one individual with two risk alleles demonstrated attenuated TNF levels (Fig. .5E). Furthermore, the release of TNF from HC macrophages in response to PAM_3_CSK_4_ did not differ significantly between wildtype, heterozygous, or homozygous individuals for *ATG16L1, IRGM*, or *IL-23R* CD-associated polymorphisms (Fig. .5F–H).

These results indicate that there is no detectable relationship between the highly significant susceptibility loci identified by GWAS and the defective release of TNF downstream of TLR activation, with the possible exception of *NOD2*, suggesting these could be independent events that coincide in patients with CD.

## DISCUSSION

We present evidence demonstrating that macrophages from CD patients have attenuated TNF release downstream of multiple TLRs. This defect was evident in all three major CD phenotypes, and was not related to use of 5-ASA medication or previous bowel inflammation. The age and gender of the patient also had no effect on the levels of TNF released. The majority of susceptibility loci that have been identified by large-scale GWAS failed to demonstrate an influence on TNF release from macrophages after TLR activation. These results build on the growing body of work supporting a defective innate immune response in CD, but highlight our lack of mechanistic insight into the cause of this abnormality. The lack of association between impaired TNF release and GWAS risk alleles also reveals that while both coexist in CD, they are likely independent. Individuals who are more likely to develop CD may have inherent susceptibility plus a weak innate immune response.

CD is a complex disease comprising multiple stages and phenotypes. We recently demonstrated that CD patients clear bacteria less rapidly than control individuals, which was associated with delayed recruitment of neutrophils and defective macrophage function.^7^ This led us to propose a “three-stage” model for CD pathogenesis—where mucosal damage and penetration of bacteria and other particulate matter from the bowel wall is followed by an impaired macrophage response and incomplete bacterial clearance. Subsequently, a compensatory adaptive immunological response develops, associated with chronic, granulomatous inflammation and an elevation in proinflammatory cytokines that is characteristic of the “active” phase of CD.^22^ Our findings here of defective TNF release by CD macrophages in response to TLR agonists is consistent with this model, given the prominent role of TNF in the acute inflammatory response, upregulation of vascular cellular adhesion molecules,^23^ and resultant recruitment of neutrophils.

A role for deficient release of TNF in the pathogenesis of CD is supported by a number of in vivo studies. The effect of TNF blockade or deficiency in the DSS murine model of colitis is particularly instructive. Although antagonists of this cytokine are highly effective at ameliorating inflammation when given to mice with established colitis, animals with a genetic deficiency of TNF are more susceptible to acute colitis than wildtype animals, with a 60% 7-day mortality compared to 0% in control animals.^24^ The dichotomous effects of this pivotal cytokine supports the concept of the “phasic” nature of CD pathogenesis and suggests that the same cytokines that have a deleterious effect during chronic inflammation may confer protection in the preceding acute inflammatory response. Polymorphisms associated with disease through GWAS are mainly markers of shared genetic regions that are present at increased frequency in individuals with disease. The biological roles these markers play remain largely unknown and to date a number of studies have been carried out on NOD2, IL23R, and ATG16L1 with contradictory results. *NOD2* polymorphisms are associated with impaired induction of proinflammatory cytokines in response to MDP,^21^ yet peripheral blood mononuclear cells from individuals with the ATG16L1 Thr300Ala CD-risk variant were recently reported to release increased levels of IL-1β after MDP stimulation,^25^ and ATG16L1 deficient macrophages secrete increased IL-1β and IL-18 in response to LPS.^26^ In this study we looked for associations between CD susceptibility polymorphisms and the defective release of TNF from macrophages. Overall, the results suggest that the strongest associated polymorphisms play no major role in the reduced TNF levels associated with macrophages from CD patients. A possible exception is *NOD2*, as CD individuals and one HC individual tested demonstrated a trend toward attenuated TNF release in response to TLR2 stimulation compared to *NOD2* wildtype individuals. Although studies conducted on macrophages from *NOD2* knockout mice revealed normal TNF release in response to TLR2 activation with peptidoglycan,^27^ further studies will be required to clarify the role of NOD2 in the human macrophage TLR2 response.

The results further suggest that the molecular basis of abnormal proinflammatory cytokine secretion by CD macrophages has not yet been identified and additional studies are needed to account for this phenomenon. A number of additional loci have recently been reported as associated with CD, several of which contain genes encoding proteins involved in vesicle and protein trafficking (*VAMP3, NDFIP1, SCAMP3*), which could have a role in proinflammatory cytokine release by macrophages. It has been estimated that ≍23% of the heritability of CD can be explained by the variants identified to date by GWAS.^4^ Furthermore, it has been postulated that rare variants, structural rearrangements, or epigenetic modifications could account for some of the “missing heritability.”^28^ It is very possible that some of these mutations, which are likely to be highly heterogeneous between individual CD patients, could be important determinants of the macrophage response to bacterial agonists.

Impairment of TNF release is more severe in patients with colonic CD. Whereas defective pathways associated with NOD2 have been implicated in the causation of ileal CD, a grossly impaired TLR response to bacteria appears to be more relevant to colonic inflammation in CD. Our results suggest that future studies on the pathogenesis of this heterogeneous condition may prove more fruitful if patients with CD are grouped according to phenotype rather than by their primary diagnosis. This is an important consideration when interpreting genome-wide association projects as well as planning therapeutic regimens and clinical trials. Work to define the precise underlying molecular defects associated with defective cytokine secretion is ongoing and may offer novel therapeutic targets in the future.

## Notes

Supporting Information Figure 1 Signalling downstream of TLR2 and the resultant pro-inflammatory gene induction are indistinguishable between CD and control macrophages. (A) Phosphorylation of p38 MAP kinase and JNK, and degradation of I?B-α in macrophages from CD (n=7) and HC (n=5) subjects in response to stimulation with PAM3. Representative western blots from individual subjects are shown on the right. (B) mRNA profiles of genes related to TLR signalling in CD (n=9) and HC (n=8) macrophages after stimulation with PAM3. There were no significant differences between macrophages from CD and HC subjects.

Supporting Information Figure 2 Supernatants from CD macrophages stimulated for 6 h with A. PAM3CSK4 and B. LPS; and the amount of TNF released was measured and correlated with age. Results for TNF against age are expressed as scatter plots with the corresponding correlation (R2).

Supporting Information Table 1 Details for the 34 CD-associated SNPs included. The potential candidate genes of interest for each locus are those reported by Franke et al. (4)
