# Supplementary figures and images for: Defective tumor necrosis factor release from Crohn's disease macrophages in response to toll-like receptor activation: Relationship to phenotype and genome-wide association susceptibility loci
Source: Inflamm Bowel Dis. 2012 Mar 20;18(11):2120–7. doi: 10.1002/ibd.22952 (PMC3532612; doi:10.1002/ibd.22952)

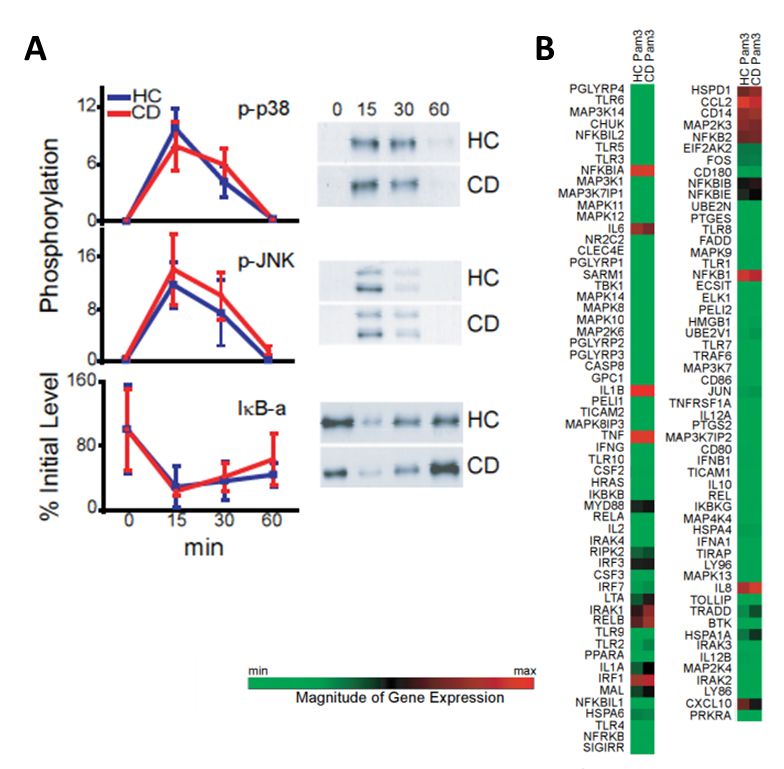

Supplement: Supporting Information Figure 1 — Signalling downstream of TLR2 and the resultant pro-inflammatory gene induction are indistinguishable between CD and control macrophages. (A) Phosphorylation of p38 MAP kinase and JNK, and degradation of I?B-α in macrophages from CD (n=7) and HC (n=5) subjects in response to stimulation with PAM3. Representative western blots from individual subjects are shown on the right. (B) mRNA profiles of genes related to TLR signalling in CD (n=9) and HC (n=8) macrophages after stimulation with PAM3. There were no significant differences between macrophages from CD and HC subjects. [file ibd0018-2120-SD1.tif]

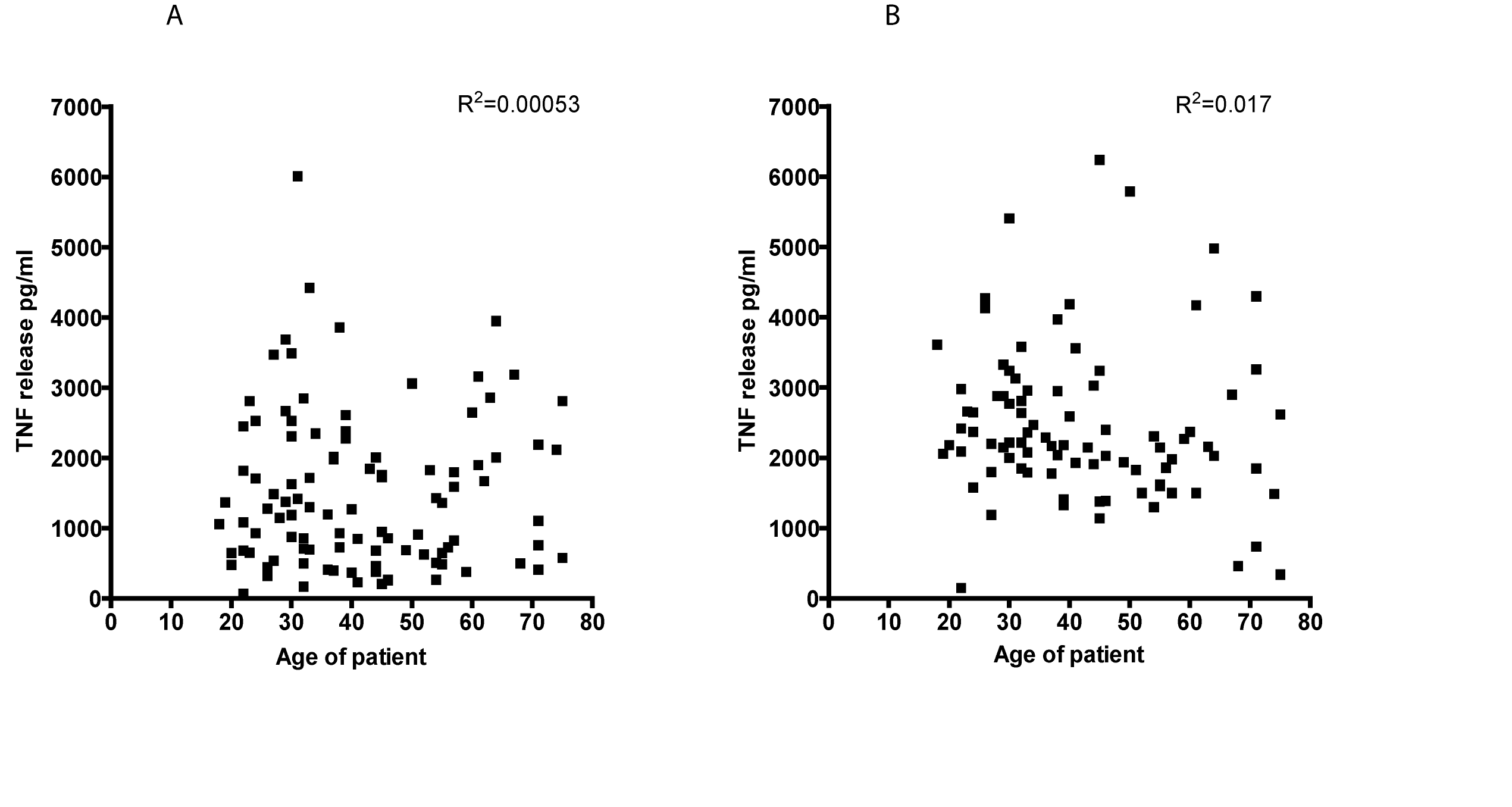

Supplement: Supporting Information Figure 2 — Supernatants from CD macrophages stimulated for 6 h with A. PAM3CSK4 and B. LPS; and the amount of TNF released was measured and correlated with age. Results for TNF against age are expressed as scatter plots with the corresponding correlation (R2). [file ibd0018-2120-SD2.tif]
