# Supplementary material for: Defective tumor necrosis factor release from Crohn's disease macrophages in response to toll-like receptor activation: Relationship to phenotype and genome-wide association susceptibility loci
Source: Inflamm Bowel Dis. 2012 Mar 20;18(11):2120–7. doi: 10.1002/ibd.22952 (PMC3532612; doi:10.1002/ibd.22952)
Supplement: Supporting Information Table 1 — Details for the 34 CD-associated SNPs included. The potential candidate genes of interest for each locus are those reported by Franke et al. (4) [file ibd0018-2120-SD3.doc]

| **dbSNP ID** | **Chromosome** | **Potential candidate genes of interest** | **Risk Allele** | **HC Frequency** | **CD Frequency** | **Odds ratio** |
| --- | --- | --- | --- | --- | --- | --- |
| rs11209026 | 1p31 | *IL23R* | G | 0.932 | 0.973 | 2.66 |
| rs9286879 | 1q24 | *TNFSF18, TNFSF4, FASLG* | G | 0.246 | 0.285 | 1.22 |
| rs11584383 | 1q32 | *C1orf106, KIF21B* | T | 0.710 | 0.733 | 1.12 |
| rs3024505 | 1q32 | *IL10, IL19* | A | 0.156 | 0.179 | 1.18 |
| rs10210302 | 2q37 | *ATG16L1* | T | 0.525 | 0.597 | 1.34 |
| rs4613763 | 5p13 | *PTGER4* | C | 0.120 | 0.163 | 1.43 |
| rs10067603 | 5q3 | *SLC22A4, SLC22A5, IRF1, IL3* | A | 0.789 | 0.821 | 1.23 |
| rs13361189 | 5q33 | *IRGM* | C | 0.086 | 0.114 | 1.37 |
| rs10045431 | 5q33 | *IL12B* | C | 0.757 | 0.792 | 1.22 |
| rs6908425 | 6p22 | *CDKAL1* | C | 0.784 | 0.805 | 1.14 |
| rs7746082 | 6q21 | *PRDM1* | C | 0.299 | 0.325 | 1.13 |
| rs2301436 | 6q27 | *CCR6* | T | 0.467 | 0.504 | 1.16 |
| rs1456893 | 7p12 | *IKZF1, ZPBP, FIGNL1* | A | 0.697 | 0.724 | 1.14 |
| rs1551398 | 8q24 |  | A | 0.610 | 0.647 | 1.17 |
| rs10758669 | 9p24 | *JAK2* | C | 0.349 | 0.387 | 1.18 |
| rs4263839 | 9q32 | *TNFSF15, TNFSF8* | G | 0.681 | 0.721 | 1.21 |
| rs17582416 | 10p11 | *CREM* | G | 0.344 | 0.374 | 1.14 |
| rs10995271 | 10p21 | *ZNF365* | G | 0.392 | 0.442 | 1.23 |
| rs1250550 | 10q22 | *ZMIZ1* | C | 0.678 | 0.710 | 1.16 |
| rs7927894 | 11q13 | *C11orf30* | T | 0.389 | 0.427 | 1.17 |
| rs11175593 | 12q12 | *MUC19, LRRK2* | T | 0.023 | 0.037 | 1.64 |
| rs3764147 | 13q14 | *C13orf31* | G | 0.245 | 0.275 | 1.17 |
| rs8049439 | 16p11 | *IL27, SH2B1, EIF3C, LAT, CD19* | C | 0.378 | 0.409 | 1.14 |
| rs2066844* | 16q12 | *NOD2* (R702W, SNP8) | T | 0.036 | 0.081 | 2.36 |
| rs2066845* | 16q12 | *NOD2* (G908R, SNP12) | C | 0.017 | 0.041 | 2.50 |
| rs2066847* | 16q12 | *NOD2* (L1007finsC, SNP13) | C | 0.022 | 0.087 | 4.24 |
| rs2872507 | 17q21 | *GSMDL, ZPBP2, ORMDL3, IKZF3* | A | 0.458 | 0.491 | 1.14 |
| rs744166 | 17q21 | *MLX, STAT3* | A | 0.583 | 0.612 | 1.13 |
| rs2542151 | 18p11 | *PTPN2* | G | 0.153 | 0.183 | 1.24 |
| rs10500264 | 19q13 |  | G | 0.807 | 0.829 | 1.16 |
| rs1736135 | 21q21 |  | T | 0.577 | 0.613 | 1.16 |
| rs762421 | 21q22 | *ICOSLG* | G | 0.383 | 0.423 | 1.18 |
| rs2412973 | 22q12 | *MTMR3* | A | 0.457 | 0.487 | 1.13 |
| rs4821544 | 22q12 | *NCF4* | C | 0.330 | 0.351 | 1.10 |

**Supporting Table 1.** Details for the 34 CD-associated SNPs included. The potential candidate genes of interest for each locus are those reported by Franke et al. (4).
